# Design, Synthesis and Sensing Application of Novel Dual Lanthanide Doped Core–Shell Fluorescent Silica-Based Nanoparticles

**DOI:** 10.3390/bios15100636

**Published:** 2025-09-24

**Authors:** Qiuping Li, Hongxia Ouyang, You Zhou, Xinghui Yang, Qi Wang, Yonghong Ding, Haichao Yu

**Affiliations:** 1 FuZhou AI Drug Innovation Center, School of Pharmacy, Fuzhou Medical University, Fuzhou 344000, China; ouyanghongxia2025@163.com (H.O.); yangxinghui8207@163.com (X.Y.); qiqidish@126.com (Q.W.); dingyonghong710909@163.com (Y.D.); yhc1411@163.com (H.Y.); 2 State Key Laboratory Base of Novel Functional Materials and Preparation Science, School of Materials Science and Chemical Engineering, Ningbo University, Ningbo 315211, China

**Keywords:** lanthanide, fluorescence, fumed silica, core–shell nanoparticles, ratio sensor

## Abstract

The synthesis of lanthanide fluorescent nanoparticles and the investigation of their fluorescence sensing applications have attracted a great deal of attention in materials science over the past decades. In this study, we designed and synthesized a core–shell fluorescent nanoparticle based on dual-center emission from the europium and terbium complexes, and demonstrated its application as a ratiometric fluorescence sensor for the detection of 2,6-pyridinedicarboxylic acid (DPA). The europium complex is embedded in the inner core, providing a stable fluorescence signal at 617 nm, while the terbium complex is positioned in the outer shell and exhibits a fluorescence “Turn-ON” response at 545 nm upon interaction with DPA molecules. The fluorescence intensity ratio F_545_/F_617_ exhibits a sensitive response to the DPA concentration. Experimental results demonstrate that the as-prepared SiO_2_@Eu@SiO_2_@Tb nanoparticle exhibits a linear response in the DPA concentration range of 10–100 μM, with a detection limit (LOD) of 1.38 μM and well selectivity for DPA sensing. This strategy offers new insights into the development of novel lanthanide-based ratiometric fluorescence sensors.

## 1. Introduction

Due to their unique optical characteristics, such as narrow emission bands, large Stokes shifts, and long fluorescence lifetimes, fluorescent sensors based on rare earth compounds have garnered significant attention [1,2,3,4,5,6,7,8,9]. Over the past few decades, a wide variety of rare earth-based fluorescent sensors have been developed and widely applied in the detection of temperature [10,11,12], metal ions [13,14,15,16], small molecules [17,18,19,20], and biomolecules [21,22,23,24], demonstrating their remarkable advantages in monitoring environmental pollutants [25,26,27,28], biomarkers [29,30,31,32], etc. To date, numerous rare earth complexes [33,34,35], nanomaterials [36,37,38,39,40,41], metal–organic frameworks (MOFs) [42,43,44,45], carbon-based materials [46,47,48,49,50], and organic–inorganic hybrid materials [51,52,53,54,55] exhibiting excellent fluorescence sensing properties have been successfully developed, particularly those incorporating Eu^3+^ and Tb^3+^ ions, which display characteristic emissions in the visible spectral region. Among these materials, silica-based rare earth hybrid materials [56,57,58,59,60] have emerged as one of the most intensively studied platforms in fluorescent sensing. This is primarily because silica-based materials are not only readily available, but also highly modifiable and easy to assemble; in addition, they offer excellent chemical stability and biocompatibility [61,62,63]. Consequently, significant research efforts have been devoted to the development of silica-based rare earth hybrid fluorescent sensing materials over the past few decades [64,65,66,67,68,69].

Most early fluorescent sensing materials relied on monitoring intensity variations from a single emission source to detect target analytes. However, this design rendered the sensor’s output highly susceptible to interference from background light, temperature fluctuations, instrumental variations, and other external factors, often resulting in distorted measurement outcomes. To address this limitation, researchers introduced an additional fluorescent emission center into the original single-emission material [70,71,72,73,74,75,76,77]. By tracking the fluctuation in the intensity ratio between the two emission centers, the system enables signal normalization and self-calibration, thereby effectively mitigating environmental and instrumental disturbances and enhancing the reliability of the sensing results [78,79,80]. Fluorescent sensors based on this strategy are referred to as ratiometric fluorescent probes. Over the years, such ratiometric probes have emerged as the preferred strategy for constructing robust sensing platforms, and a wide variety of ratiometric fluorescent probes have been developed to date [81,82,83,84,85,86,87,88,89,90,91,92,93]. Compared with other fluorescent materials, lanthanide-based fluorescent sensors typically exhibit fixed, sharp emission peaks, which facilitate accurate identification. Owing to these distinct advantages, ratiometric fluorescent probes incorporating two or more lanthanide ions have unique advantages. As early as 2007, Tremblay et al. [94] reported a ratiometric probe based on a heterometallic Tb^3+^/Eu^3+^ bis-lanthanide chelate capable of responding to chemical and environmental variations. More recently, Li et al. [95] developed a ratiometric fluorescent sensor utilizing a bimetallic lanthanide metal–organic framework, which enables the precise detection of Hg^2+^ ions. Although significant progress has been made in the development of multi-lanthanide ratiometric fluorescent sensors, this field still offers substantial potential for further exploration [96,97].

Bacillus anthracis is a pathogenic bacterium that can cause severe infectious diseases in both humans and animals [98]. Due to its high toxicity and significant lethality, its spores have the potential to be exploited as biochemical warfare agents, posing a serious threat to public health and global security. Therefore, the development of rapid and accurate detection technologies for monitoring Bacillus anthracis spores is of critical importance in preventing disease outbreaks and mitigating the risks of bioterrorism. Pyridine-2,6-dicarboxylic acid (DPA), a major constituent of Bacillus anthracis spores, has long been recognized as a key biomarker for its detection [99]. When environmental concentrations of DPA exceed 60 μM [100,101,102], increased vigilance is warranted to address the potential risk of Bacillus anthracis infection. Among the various methods available for detecting DPA, fluorescence analysis—particularly the ratio-based fluorescence sensing method—has garnered significant attention due to its high sensitivity, rapid response, and operational simplicity. For example, Xu et al. [103] reported a kind of lanthanide-based metal–organic framework material that could detect DPA through ratiometric fluorescent and electrochemiluminescent dual-mode. The detection limit of the ratiometric fluorescent sensing function of this material for DPA molecules is 6.8 × 10^−10^ M. To date, numerous ratiometric fluorescent sensors based on lanthanide luminescence have been developed for the detection of DPA [104,105,106].

In this study, we employed a commercialized silicon-based material, silica fume—a type of silica microsphere synthesized via the gas-phase method—as the carrier. Utilizing the molecular bridging capability of silane coupling agents [107,108,109,110], rare earth complexes were successfully grafted onto its surface, leading to the development of a kind of novel multi-emission fluorescent microsphere with a multi-layered core–shell architecture. Furthermore, we systematically investigated the structural characteristics, photophysical properties, and potential applications of this material in fluorescent sensing of DPA.

## 2. Materials and Methods

### 2.1. Materials and Testing Instruments

Tetraethyl orthosilicate (TEOS, 98%), EuCl_3_·6H_2_O (99.9%, metals basis), TbCl_3_·6H_2_O (99.9%, metals basis), and fumed silica (SiO_2_, 99%, metals basis) was purchased from Aladdin Biochemical Technology (Shanghai, China). 3-aminopropyltriethoxysilane (APTS, 98%) was purchased from Biosharp Technology (Hefei, China). 1,10-phenanthrolin-5-amine (phen-NH_2_, 98.04%) was provided by Bide Pharmatech (Shanghai, China). 2-thenoyltrifluoroacetone (TTA, 99%) was provided by Maya Reagent (Jiaxing, China). Diethylenetriaminepentaacetic (DTPA) dianhydride (97%) was provided by Shanghai Leyan (Shanghai, China). 2,6-pyridinedicarboxylic acid (DPA, 99%), benzoic acid (BA, 99%), L-ascorbic acid (VC, 99%), acetic acid (AA, 99.5%), valine (Val, 99%), tyrosine (Tyr, 99%), leucine (Leu, 99%), glutamic acid (Glu, 99%), proline (Pro, 99%), and 3-isocyanatopropyltriethoxysilane (ICPTS, 95%) were provided by Macklin Biochemical Technology (Shanghai, China). The anhydrous toluene was prepared by distillation after dehydrating the corresponding reagent with magnesium sulfate. Ammonia, anhydrous ethanol, petroleum ether, NaCl, KCl, and all other reagents used in this study were of analytical grade and were purchased from Sinopharm Chemical Reagent (Shanghai, China).

Fourier transform infrared spectroscopy (FTIR) was tested with an Agilent Cary 630 (Agilent Technologies, Santa Clara, CA, USA). Ultraviolet–visible (U–vis) absorption was characterized on a Yoke T-N6000 Plus (Shanghai Yoke Instrument Co., Ltd., Shanghai, China). The fluorescent emission spectra, fluorescent excitation spectra and photometric value were measured with a Gangdong F-320 (Tianjin Gangdong SCI.&TECH Co., Ltd., Tianjin, China). Thermogravimetric analysis (TGA) was performed using a Netzsch STA 449 F3 instrument (Netzsch, Berlin, Germany) under a protective N_2_ atmosphere. Transmission electron microscope (TEM) images were taken using a Thermo Fisher Talos F200S G2 (Thermo Fisher, Brno, Czech Republic), as were the energy dispersive spectrometer (EDS) spectra. The ultrasonic instrument employed in the experiment was a Derui DR-M10 (Shenzhen Derui Ultrasonic Equipment Co., Ltd., Shenzhen, China), featuring a chamber volume of 1 L, an ultrasonic frequency of 40 kHz, and a maximum output power of 80 W.

### 2.2. Preparation of Eu(TTA)_3_(H_2_O)_2_

The Eu(TTA)_3_(H_2_O)_2_ was synthesized according to an improved literature method [111]. First, we dissolved TTA and EuCl_3_·6H_2_O in a mixed solvent of ethanol and water at a molar ratio of 3:1. The mixed solution was adjusted to pH ≈ 7 using ammonia water and then subjected to reflux for 1 h. After that, the majority of the ethanol was removed through evaporation. The cooled solution was then filtered to collect the precipitate. Subsequently, the crude product was fully dispersed in 30 mL petroleum ether and filtered again, with the residue being washed repeatedly with petroleum ether during this step. Finally, the product was dried under vacuum at 55 °C, yielding a pale-yellow powder identified as Eu(TTA)_3_(H_2_O)_2_.

### 2.3. Surface Modification of Fumed Silica with Europium Complexes

First, the ligand phen-NH_2_ was covalently bonded to the surface of fumed silica using silane coupling agent. As is typical, ICPTS and phen-NH_2_ were reacted in a 1:2.5 molar ratio in anhydrous DMF at room temperature for 8 h. Subsequently, the resulting reaction mixture was combined with an anhydrous toluene dispersion of fumed silica. The mixture was then stirred under water-isolated conditions at 100 °C for 24 h. After that, the product was collected via filtration and washed alternately with dichloromethane and ethanol. The product was dried under vacuum at 55 °C and identified as SiO_2_-phen.

Secondly, SiO_2_-phen was dispersed into the ethanol solution of Eu(TTA)_3_(H_2_O)_2_, and the chelation reaction was carried out at room temperature for 24 h. The product was subsequently collected via filtration, washed thoroughly with ethanol, and dried under vacuum at 55 °C. The resulting material was designated as SiO_2_@Eu.

### 2.4. Preparation of Core–Shell SiO_2_@Eu@SiO_2_@Tb Nanoparticles

The coating of SiO_2_@Eu was performed following the modified Stöber method [112,113,114]. Typically, A total of 300 mg of SiO_2_@Eu was ultrasonically dispersed in 50 mL ethanol. Under vigorous stirring, 1 g of TEOS, 10 mL distilled water and 0.5 mL ammonia were added sequentially to the reaction system. The mixture was then stirred vigorously at 40 °C for 8 h. The product was collected via centrifugation, subsequently redispersed in 30 mL deionized water for washing, and subjected to repeated centrifugation. This centrifugation–washing–centrifugation procedure was performed five times to ensure complete removal of impurities. Finally, the purified product was dried at 55 °C, yielding SiO_2_@Eu@SiO_2_.

The SiO_2_@Eu@SiO_2_ was then dispersed in 10 mL anhydrous toluene, followed by the addition of 1 mL APTS. The mixture was stirred at 115 °C under water-isolated conditions for 12 h. Subsequently, the product was collected via filtration and washed alternately with dichloromethane and anhydrous ethanol three times. Finally, the material was vacuum-dried at 55 °C to yield SiO_2_@Eu@SiO_2_@APTS.

Then, SiO_2_@Eu@SiO_2_@APTS was dispersed in 20 mL anhydrous toluene, an anhydrous toluene solution containing 300 mg DTPA was introduced, along with 0.25 mL glacial acetic acid serving as a catalyst. The reaction mixture was then maintained at 80 °C under continuous stirring for 6 h. The product was collected via filtration and washed alternately with ethanol and enough deionized water. The material was also vacuum-dried at 55 °C to yield SiO_2_@Eu@SiO_2_@DTPA.

Finally, SiO_2_@Eu@SiO_2_@DTPA was dispersed into the ethanol solution of TbCl_3_·6H_2_O, stirring at room temperature for 24 h. The product was collected via filtration, washed thoroughly with ethanol, and dried under vacuum at 55 °C. The final product was designated as SiO_2_@Eu@SiO_2_@Tb.

### 2.5. Sensing Experiments

In a typical experimental procedure, 50 mg of SiO_2_@Eu@SiO_2_@Tb is first dispersed into a sealed tube containing 50 mL of deionized water, and then ultrasonicated for 30 min to ensure uniform dispersion. To ensure and maintain the uniformity of the SiO_2_@Eu@SiO_2_@Tb aqueous dispersion throughout the experimental process, continuous ultrasonic treatment is required.

The testing protocol for the sensing selectivity of SiO_2_@Eu@SiO_2_@Tb is as follows: A series of tubes is prepared by sequentially adding aqueous solution of Cl^−^, CO_3_^2−^, HPO_4_^2−^, Na^+^, K^+^, Ca^2+^, Mg^2+^, BA, VC, AA, Val, Tyr, Leu, Glu, Pro, DPA and water. Subsequently, an appropriate volume of SiO_2_@Eu@SiO_2_@Tb aqueous dispersion is introduced into each tube to achieve final concentrations of 50 μM for each control substance and DPA, and 0.5 mg/mL for SiO_2_@Eu@SiO_2_@Tb. Following sealing, the tubes are ultrasonicated for 5 min. We visually examined the fluorescence of each tube under a 254 nm UV lamp.

The evaluation of the sensing performance of SiO_2_@Eu@SiO_2_@Tb for DPA was conducted as follows: A series of sensing test solutions was prepared following a procedure analogous to the one previously described. Each tube contained 0.5 mg/mL SiO_2_@Eu@SiO_2_@Tb and varying concentrations of DPA (10, 20, 30, 40, 50, 60, 70, 80, 90, and 100 μM). After sealing, the samples were ultrasonicated for 5 min, and their fluorescence emission properties were subsequently measured.

## 3. Results and Discussion

**Scheme 1:**
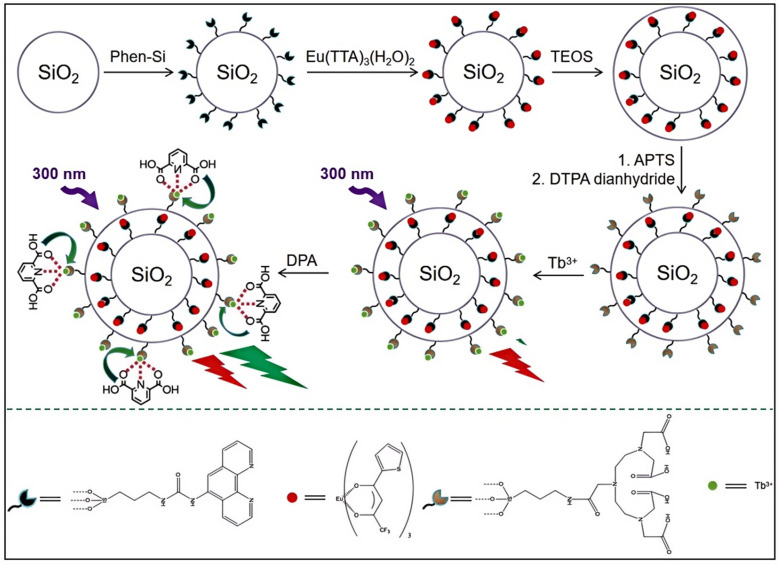
The design and synthesis method of a lanthanide-based core–shell ratio fluorescent sensor.

Most silicon nanoparticles employed in research are synthesized using the modified Stöber method. In this paper, we present a novel approach in which commercially available gas-phase-synthesized nano-silica (Fumed Silica) is directly utilized as a nano-carrier. By employing a layer-by-layer assembly strategy, we have successfully fabricated a new dual-emission fluorescent nanoparticle (SiO_2_@Eu@SiO_2_@Tb). Scheme 1 illustrates the design and synthesis methodology of this innovative lanthanide-based core–shell fluorescent nanoparticle. First, we introduced a phen moiety onto the surface of commercially obtained silica spheres via ICPTS; subsequently, a layer of europium complex was adsorbed on to the silica surface via the coordination ability of the phen moiety. Second, a modified Stöber method was employed to coat the SiO_2_@Eu core, effectively embedding the europium complex within the silica shell to obtain SiO_2_@Eu@SiO_2_. Third, on the outer surface of the newly formed silica shell, a silane coupling agent (APTS) was used to tether a DTPA molecule, which served as a chelating agent for Tb^3+^ ions. Finally, a multi-layered core–shell structured nanoparticle was successfully constructed. In the nanoparticle, the europium complex embedded within the SiO_2_ shell serves as a stable source of fluorescence signal, whereas the terbium complex located on the outer layer initially exhibits only a very weak characteristic emission. Notably, upon interaction with DPA molecules, the fluorescence signal originating from the Tb^3+^ ions demonstrates a distinct “turn-on” response.

**Figure 1:**
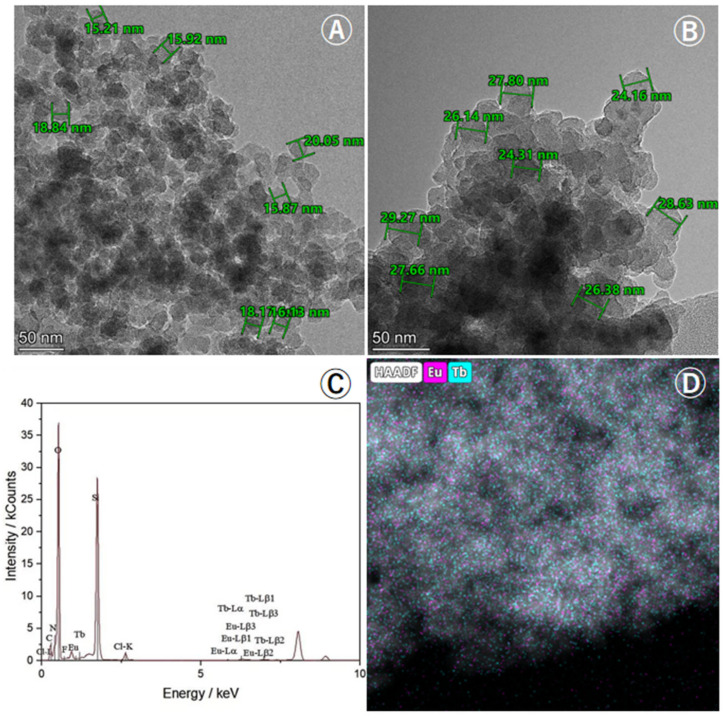
TEM images of the raw material ((**A**), SiO_2_) and final core–shell nanoparticles ((**B**), SiO_2_@Eu@SiO_2_@Tb); EDS (**C**) and HAADF (**D**) image of the final core–shell nanoparticles. (For detailed high-definition large images, please refer to the Supplementary Materials).

The particle size changes before and after the nano-silica coating process can be observed in Figure 1A,B. Despite the significant agglomeration of nano-silica during TEM analysis, the original SiO_2_ microspheres (fumed silica) in Figure 1A exhibit a particle size of approximately 20 nm. Following the multilayer assembly to form SiO_2_@Eu@SiO_2_@Tb, the particle size increased by approximately one third, demonstrating the feasibility of our coating strategy based on the modified Stöber method. EDS analysis of SiO_2_@Eu@SiO_2_@Tb (Figure 1C) confirmed that both Eu^3+^ and Tb^3+^ ions had been incorporated into the final core–shell nanoparticles. However, as the doping process relies on surface modification and coordination-based assembly, the concentrations of Eu^3+^ and Tb^3+^ ions within the final nanoparticles remained relatively low, resulting in the weak corresponding signals shown in Figure 1C. Furthermore, the HAADF image of SiO_2_@Eu@SiO_2_@Tb was also conducted, and the elemental distribution maps for Eu and Tb elements are presented in Figure 1D. The uniform distribution of both elements across the entire material further supports the successful incorporation of Eu^3+^ and Tb^3+^ ions into the nano-silica matrix. The loading of the rare earth complex within the core–shell nano-silica can also be inferred from its TG analysis curve. As shown in Figure S1, SiO_2_@Eu@SiO_2_@Tb exhibits three possible weight loss stages between 210 °C and 750 °C. The first stage (210 °C to 382 °C), with a mass loss of approximately 3.89%, and the second stage (382 °C to 455 °C), with a loss of about 2.96%, can be attributed to the thermal decomposition of the outer terbium complex layer and the embedded europium complex layer, respectively. The third stage, characterized by a more significant weight loss of approximately 7.87%, may result from the continuous pyrolysis of the rare earth compounds and the silane coupling agent on the surface of the nano-silica spheres under high-temperature conditions. These TG observations provide further reliable evidence for the successful incorporation of rare earth complexes into the nano-silica matrix.

**Figure 2:**
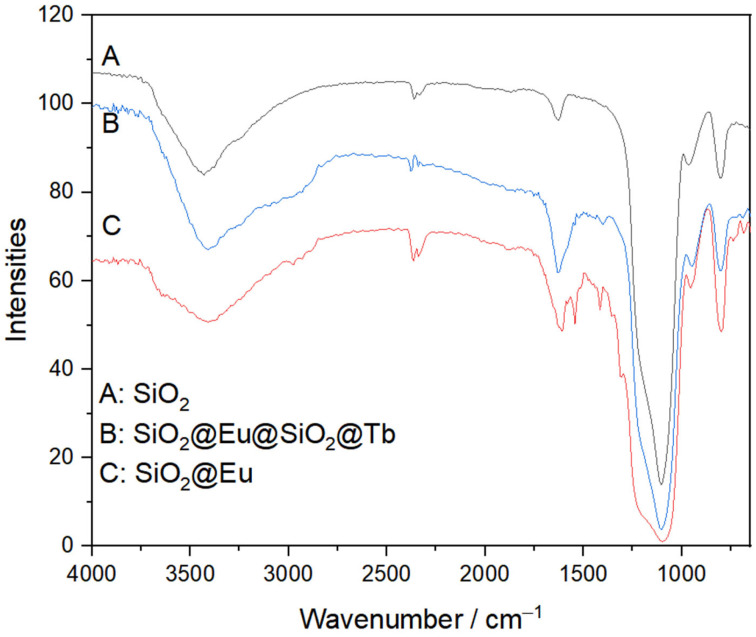
FTIR spectra of SiO_2_ (A), SiO_2_@Eu (C) and SiO_2_@Eu@SiO_2_@Tb (B).

Figure 2 presents the FT-IR spectra of (A) fumed silica (SiO_2_), (B) SiO_2_@Eu@SiO_2_@Tb, and (C) SiO_2_@Eu. As shown in Figure 2 in A, the absorption features observed for fumed silica originate from the framework vibrations of silicon-based materials. Specifically, the characteristic peaks at 805 cm^−1^ and 1103 cm^−1^ correspond to the symmetric (ν_s_) and antisymmetric (ν_as_) stretching vibrations of Si–O–Si bonds, respectively, whereas the peak at 969 cm^−1^ is attributed to the stretching vibration of Si–OH groups. Figure 2 in C presents the FT-IR spectrum of SiO_2_@Eu. Compared with fumed silica, the spectrum of SiO_2_@Eu exhibits several distinct changes: (1) A series of weak absorption peaks emerge between 3100 cm^−1^ and 2800 cm^−1^, which can be attributed to the stretching vibrations of C–H bonds in the methyl and methylene groups of the moieties of the silane coupling agent or the europium complex; (2) a strong absorption peak appears at 1610 cm^−1^, corresponding to the stretching vibration of the carbonyl group (C=O) in the coordination structure; (3) another prominent peak at 1543 cm^−1^ is assigned to the stretching vibration of the C=N bond in the phen moieties; finally, (4) absorption bands at 1416 cm^−1^ and 1357 cm^−1^ are observed, which are associated with the stretching vibrations of C=C and C=S bonds in the thiophene ring structure [115]. Figure 2 in B shows spectra of the final core–shell nanoparticles (SiO_2_@Eu@SiO_2_@Tb). Compared with SiO_2_@Eu, the FT-IR spectrum of SiO_2_@Eu@SiO_2_@Tb exhibits a reduction in several characteristic peaks, with a prominent broad absorption band emerging between 1730 cm^−1^ and 1530 cm^−1^. This observation may be attributed to two main factors: first, a partial loss of europium complexes occurs during the SiO_2_ shell coating process; second, the relative content of europium complexes decreases after the formation of the core–shell structure. Concurrently, the (C=O) stretching vibration from the carbonyl groups present in the Tb-DTPA moieties, which is adsorbed on the surface of the final product SiO_2_@Eu@SiO_2_@Tb, overlaps and couples with other vibrational modes within this range, resulting in the formation of a single, intense absorption peak. Collectively, these newly observed absorption features provide clear evidence that the europium/terbium complexes have been successfully doped into the core–shell nanoparticles.

**Figure 3:**
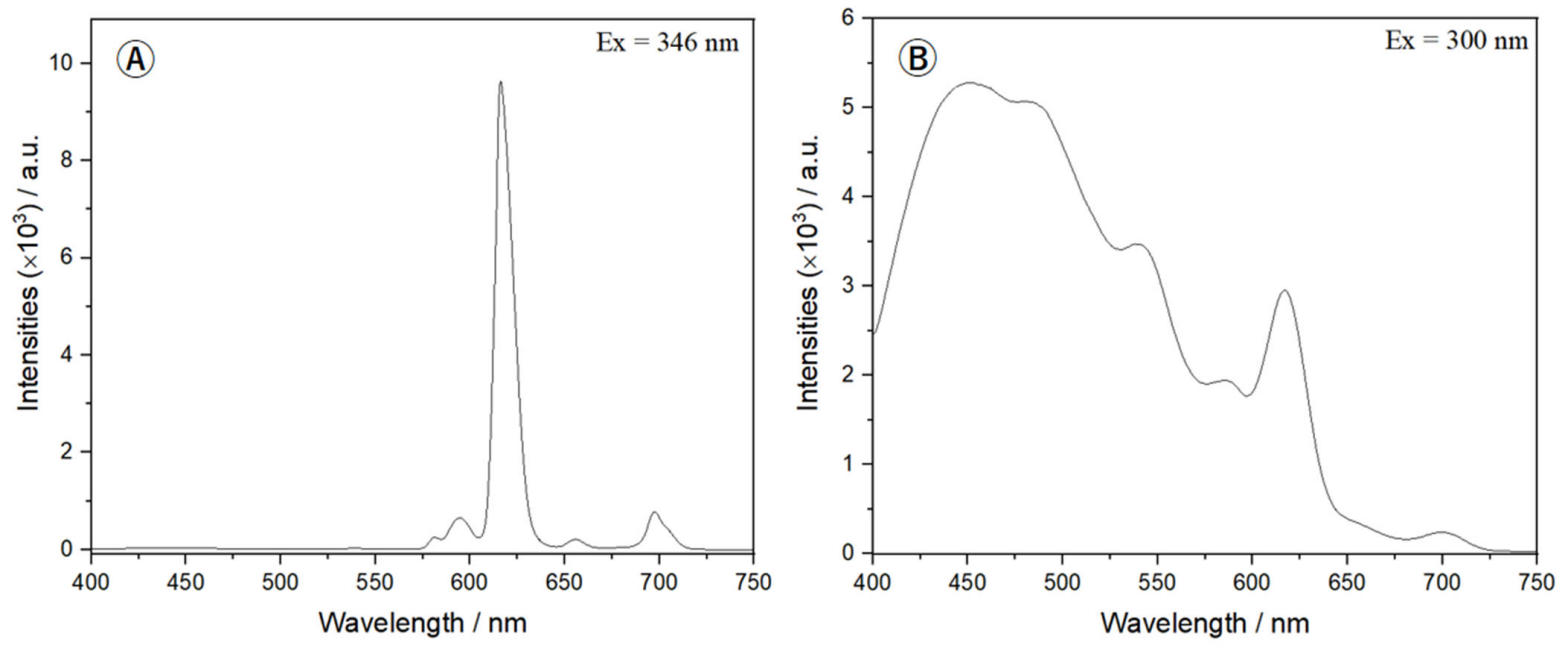
Fluorescence emission spectra of SiO_2_@Eu (**A**) and SiO_2_@Eu@SiO_2_@Tb (**B**).

Figure 3A displays the fluorescence emission spectrum of SiO_2_@Eu, which was recorded by exciting the sample at its optimal excitation wavelength of 346 nm. Clearly, this represents a characteristic fluorescence emission spectrum of Eu^3+^ ions, where the most intense emission peak around 617 nm corresponds to the ^5^D_0_ → ^7^F_2_ transition. The fluorescence emission spectrum of SiO_2_@Eu@SiO_2_@Tb is presented in Figure 3B. To achieve simultaneous and effective excitation of both Eu^3+^ and Tb^3+^ ions, an excitation wavelength of 300 nm was selected. Because a partial loss of europium complexes occurs during the SiO_2_ shell coating process, there is a reduced fluorescence emission from the Eu^3+^ ions. Nevertheless, its characteristic emission peak at 617 nm remains clearly discernible. In contrast, for the Tb^3+^ ions chelated on the surface, the fluorescence emission is significantly weaker due to the limited sensitization efficiency of DTPA toward Tb^3+^ ions. Consequently, the fluorescent emission of Tb^3+^ ions is nearly obscured by the background fluorescence of the silicon-based matrix. It should be noted that, in SiO_2_@Eu, due to the strong luminescence performance of the europium complexes loaded on the surface of the silica, the background fluorescence of the silicon-based matrix is suppressed, so no obvious background emission can be seen in Figure 3A.

**Figure 4:**
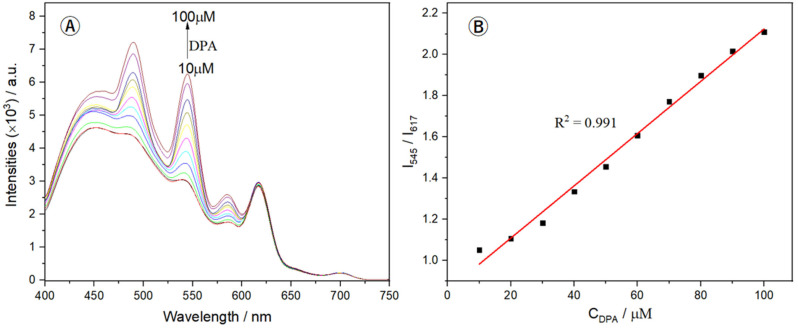
Fluorescence emission spectra of SiO_2_@Eu@SiO_2_@Tb (**A**) with different concentrations of DPA and the fitting curve (**B**) of the fluorescence intensity ratio at 545 nm and 617 nm.

As illustrated in the design schematic of Scheme 1, the europium complexes are embedded within the SiO_2_ shell, resulting in a relatively stable fluorescence emission signal from the Eu^3+^ ions that is resistant to interference from environmental components. In contrast, the terbium complexes are located on the outermost layer and are therefore susceptible to fluorescence signal variations caused by external substances. Owing to extensive studies confirming that DPA molecules can effectively sensitize the luminescence of Tb^3+^ and Eu^3+^ ions [116,117,118], and given that DPA can serve as a specific biomarker for *Bacillus anthracis*, the development of fluorescent sensors capable of the rapid and quantitative detection of DPA has long remained a central focus in the field of fluorescence sensing [119,120,121,122]. Therefore, DPA molecules were selected as the target analyte to evaluate the ratiometric fluorescence sensing capability of SiO_2_@Eu@SiO_2_@Tb. First, the UV–vis absorption spectra of the SiO_2_@Eu@SiO_2_@Tb solution were recorded in both the absence and presence of DPA. As shown in Figure S2, the spectra of SiO_2_@Eu@SiO_2_@Tb–DPA exhibit an additional absorption peak at approximately 273 nm compared with those of pure SiO_2_@Eu@SiO_2_@Tb, which can be regarded as an indicator that DPA has coordinated with the Tb^3+^ ions [123]. The triplet state (T_1_) of the DPA ligand is higher than the resonant energy level (^5^D_4_) of the Tb^3+^ ion (Δ_T1-5D4_ ≈ 5100 cm^−1^), enabling it to function as an efficient sensitizer that enhances the fluorescence of the Tb^3+^ ion and thereby generates strong green fluorescence upon ultraviolet excitation. Furthermore, driven by both binding affinity and thermodynamic factors, DPA preferentially coordinates with the Tb^3+^ ion, displacing inner-sphere H_2_O molecules and thereby suppressing the non-radiative energy transfer processes. These mechanisms collectively account for the remarkable sensitivity and selectivity of terbium-based fluorescent probes toward DPA [124,125,126]. Subsequently, the fluorescence responses of SiO_2_@Eu@SiO_2_@Tb to varying concentrations of DPA were measured under 300 nm excitation and displayed in Figure 4A. As illustrated in the figure, the emission peaks corresponding to the ^5^D_4_ → ^7^F_6_, ^5^D_4_ → ^7^F_5_, and ^5^D_4_ → ^7^F_4_ transitions of Tb^3+^ ions gradually increase as the DPA concentration rises from 10 to 100 μM. In contrast, the emission peak intensity associated with the ^5^D_0_ → ^7^F_2_ transition of Eu^3+^ ions remains nearly constant. Among all of the emission peaks of Tb^3+^ ions, the ^5^D_4_ → ^7^F_5_ (around 545 nm) transition typically exhibits the highest intensity. By designating the fluorescence signal from the ^5^D_4_ → ^7^F_5_ transition as the response signal and that from the ^5^D_0_ → ^7^F_2_ transition as the reference signal, the SiO_2_@Eu@SiO_2_@Tb system can function as a ratiometric fluorescent probe for detecting DPA. The fluorescence intensity ratio F_545_/F_617_ demonstrates a strong linear relationship with DPA concentration within the range of 10–100 μM (Figure 4B), following the regression equation F_545_/F_617_ = 0.856 + 0.013 × c(DPA) (R^2^ = 0.991). The limit of detection (LOD) was calculated as 1.38 μM using the threefold standard deviation method (3σ/slope—the relevant data of σ are presented in Table S1), which is significantly lower than the previously reported infectious dose of bacterial spores (60 μM) [100,101,102]. Overall, the remarkably wide linear response range and low limit of detection (LOD) render this core–shell nanoparticles as a viable alternative fluorescent probe for the determination of DPA concentration.

**Figure 5:**
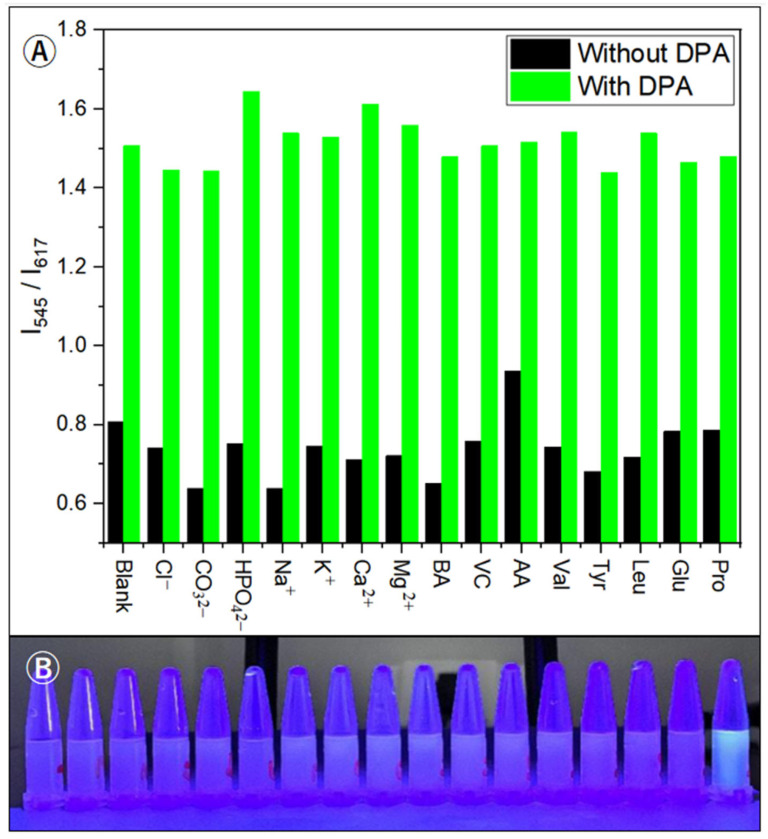
(**A**) Fluorescence responses of the SiO_2_@Eu@SiO_2_@Tb in the absence and presence of 50 μM interfering substance. (**B**) A color photo of SiO_2_@Eu@SiO_2_@Tb solutions mixed with various substances under 254 nm UV light, which are as follows, from left to right: blank control, Cl^−^, CO_3_^2−^, HPO_4_^2−^, Na^+^, K^+^, Ca^2+^, Mg^2+^, BA, VC, AA, Val, Tyr, Leu, Glu, Pro and DPA.

To further evaluate the anti-interference capability and selectivity of SiO_2_@Eu@SiO_2_@Tb as a ratiometric fluorescent sensor, we conducted comparative experiments using a series of common potential interferents, including Cl^−^, CO_3_^2−^, HPO_4_^2−^, Na^+^, K^+^, Ca^2+^, Mg^2+^, benzoic acid (BA), L-ascorbic acid (VC), acetic acid (AA), valine (Val), tyrosine (Tyr), leucine (Leu), glutamic acid (Glu) and proline (Pro). As shown in Figure 5A, when the interfering substances were introduced individually, the fluorescence intensity ratio (F_545_/F_617_) of SiO_2_@Eu@SiO_2_@Tb exhibited only minor variations that were statistically insignificant compared with the blank control. Similarly, in the presence of 50 μM DPA, the change in fluorescence intensity ratio (F_545_/F_617_) relative to the blank control remained negligible. Furthermore, a color photo of the SiO_2_@Eu@SiO_2_@Tb solutions mixed with various substances under 254 nm UV light were taken and is displayed in Figure 5B. Clearly, only DPA was capable of sensitizing SiO_2_@Eu@SiO_2_@Tb to emit visible green fluorescence upon excitation. In contrast, under identical experimental conditions, no observable green fluorescence was detected when the SiO_2_@Eu@SiO_2_@Tb solution was mixed with other interfering substances. The capability of this probe to generate a visually discernible signal via a “Turn-On” mechanism using only a cheap UV lamp facilitates its application in the rapid qualitative detection of potential DPA in resource-limited areas.

## 4. Conclusions

In conclusion, we designed and synthesized a novel core–shell fluorescent nanoparticle doped with dual lanthanide ions, Eu^3+^ and Tb^3+^, which exhibits characteristic intensity ratio variation in fluorescence emission peaks and demonstrates effective application as a fluorescent probe for DPA. The nanoparticle designed and synthesized in this article exhibits several advantageous properties. First, the starting material is readily available fumed silica, and the overall matrix is a biocompatible silicon material. Second, the reference signal originates from the europium complex embedded within the inner layer and encapsulated by a SiO_2_ shell, ensuring stability and clarity. Third, analytical results demonstrate that the nanoparticle achieves a broad linear detection range (10–100 µM), a low limit of detection (1.38 μM), and excellent selectivity for DPA sensing. Finally, this fluorescent probe could generate a visually detectable “Turn-On” signal upon interaction with a specific concentration of DPA molecules, enabling its application in the rapid qualitative detection of DPA in resource-limited settings. Certainly, this research still offers ample opportunities for further exploration. For example, strategies to minimize the loss of the europium complex during the encapsulation process and approaches to further improve the sensitivity of DPA sensing represent promising directions for future investigation. In conclusion, this study reports a novel dual-lanthanide-doped ratiometric fluorescence sensor, which not only expands the repertoire of DPA sensing platforms but also provides new insights into the design of ratiometric fluorescence sensors.

## Data Availability

The original contributions presented in this study are included in the article/Supplementary Material. Further inquiries can be directed to the corresponding authors.

## Supplementary Materials

The following supporting information can be downloaded at https://www.mdpi.com/article/10.3390/bios15100636/s1, Figure S1 TG analysis curve of the final core–shell nanoparticles; Figure S2 UV-vis absorption spectra of SiO_2_@Eu@SiO_2_@Tb and SiO_2_@Eu@SiO_2_@Tb-DPA; Table S1: The standard deviation data observed in the fluorescent probe measurements of SiO_2_@Eu@SiO_2_@Tb during the analysis of the blank sample.
