# Supplementary material for: Design, Synthesis and Sensing Application of Novel Dual Lanthanide Doped Core–Shell Fluorescent Silica-Based Nanoparticles
Source: Biosensors (Basel). 2025 Sep 24;15(10):636. doi: 10.3390/bios15100636 (PMC12562495; doi:10.3390/bios15100636)
Supplement: Supplementary file 1 [file biosensors-15-00636-s001.zip › biosensors-3855981-supplementary.pdf]

biosensors

MDPI

Supplementary materials

Design, Synthesis and Sensing Application of Novel Dual Lanthanide Doped Core–Shell Fluorescent Silica-Based Nanoparticles

Qiuping Li [1,*], Hongxia Ouyang [1], You Zhou [2,*], Xinghui Yang [1], Qi Wang [1], Yonghong Ding [1] and Haichao Yu [1]

[1] FuZhou AI Drug Innovation Center, School of Pharmacy, Fuzhou Medical University, Fuzhou 344000, China; ouyanghongxia2025@163.com (H.O.); yangxinghui8207@163.com (X.Y.); qiqidish@126.com (Q.W.); dingyonghong710909@163.com (Y.D.); yhc1411@163.com (H.Y.)

[2] State Key Laboratory Base of Novel Functional Materials and Preparation Science, School of Materials Science and Chemical Engineering, Ningbo University, Ningbo 315211, China

* Correspondence: liqiuping@yeah.net (Q.L.); zhouyou@nbu.edu.cn (Y.Z.)

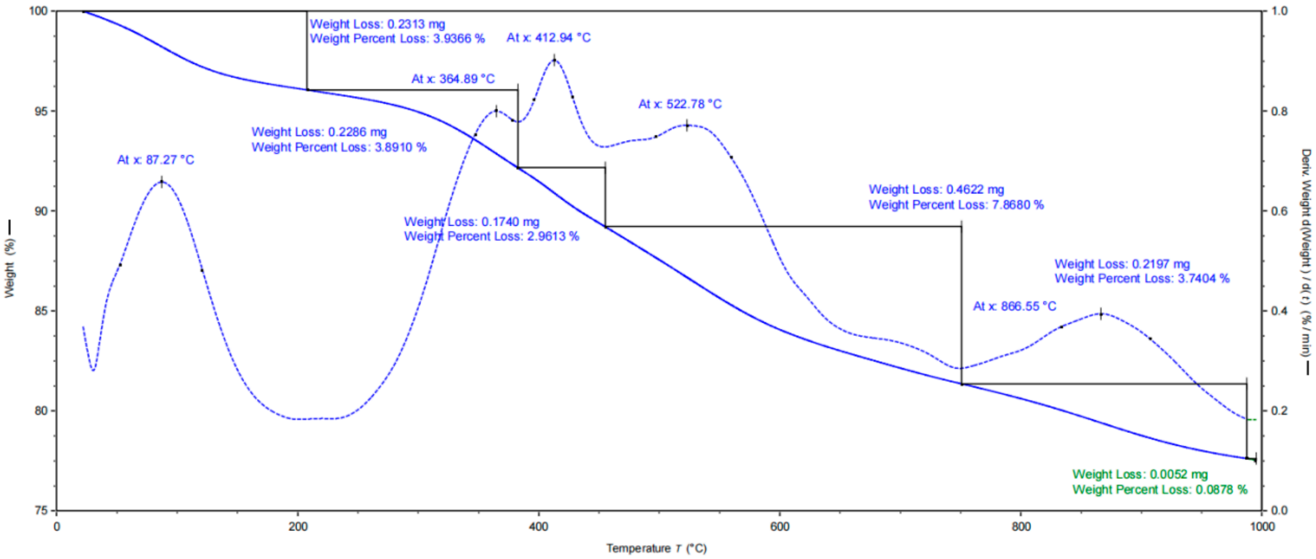

Figure S1. TG analysis curve of the final core–shell nanoparticles.

 https://doi.org/10.3390/bios15100636

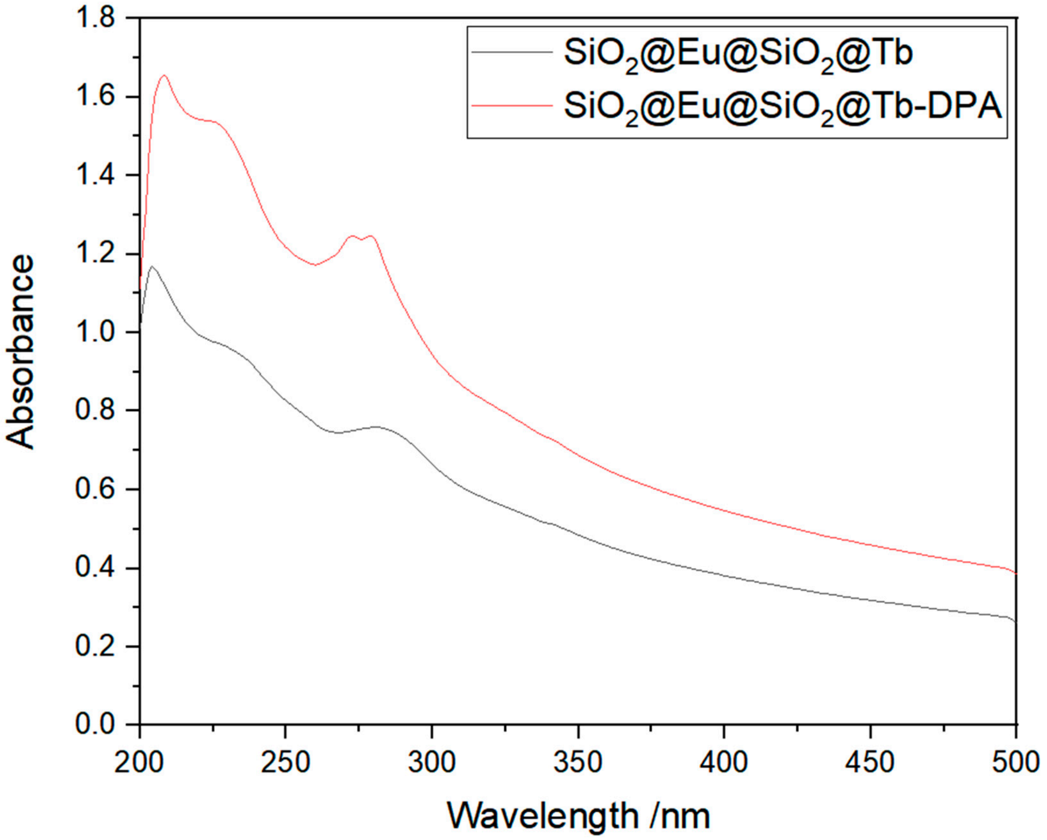

Figure S2. UV-Vis absorption spectra of $SiO_2@Eu@SiO_2@Tb$ and $SiO_2@Eu@SiO_2@Tb$-DPA.

The preparation process of $SiO_2@Eu@SiO_2@Tb$-DPA is as follows: A solution containing 0.5 mg/mL $SiO_2@Eu@SiO_2@Tb$ and 50 μM DPA was left to react for 10 minutes, then $SiO_2@Eu@SiO_2@Tb$-DPA was collected by centrifugation and wash with deionized water repeatedly.

Table S1. The standard deviation data observed in the fluorescent probe measurements of $SiO_2@Eu@SiO_2@Tb$ during the analysis of blank sample.

**Number**	**1**	**2**	**3**	**4**	**5**	**6**	**7**	**8**	**9**	**10**	**11**
I_{545}/I_{617}	1.070	1.065	1.057	1.062	1.058	1.070	1.061	1.072	1.063	1.058	1.070
	12	13	14	15	16	17	18	19	20	Average	σ
	1.057	1.064	1.060	1.054	1.050	1.055	1.065	1.059	1.061	1.062	0.006
